# A microparticle delivery system for extended release of all-trans retinoic acid and its impact on macrophage insulin-like growth factor 1 release and myotube formation

**DOI:** 10.1016/j.ijpharm.2024.124821

**Published:** 2024-10-11

**Authors:** Candice V. Cheung, Kidochukwu J. Atube, Nicholas A. Colonna, Griffin J. Carter, Tristan Marchena, Samantha McCarthy, Kelsey E. Krusen, Richard S. McCain, Norma Frizzell, R Michael Gower

**Affiliations:** a Biomedical Engineering Program, University of South Carolina, Columbia, SC 29208, USA; b Department of Chemical Engineering, University of South Carolina, Columbia, SC 29208, USA; c Department of Pharmacology, Physiology & Neuroscience, School of Medicine, University of South Carolina, Columbia, SC 29209, USA; d Veterans Affairs Medical Center, Columbia, SC 29209, USA

**Keywords:** Atrophy, Drug delivery, Macrophage, Regeneration, Retinoic acid, Skeletal muscle

## Abstract

Muscle atrophy secondary to disuse, aging, or illness increases the risk of injury, prolonged recovery, and permanent disability. The recovery process involves macrophages and their secretions, such as insulin-like growth factor 1 (IGF-1), which direct muscle to regenerate and grow. Retinoic acid receptor (RAR) activation in macrophages increases IGF-1 expression and can be achieved with all-trans retinoic acid (ATRA). However, poor bioavailability limits its clinical application. Thus, we encapsulated ATRA into poly(lactide-co-glycolide) microparticles (ATRA-PLG) to maintain bioactivity and achieve extended release. ATRA-PLG induces IGF-1 release by RAW 264.7 macrophages, and conditioned media from these cells enhances C2C12 myotube formation through IGF-1. Additionally, ATRA released from ATRA-PLG enhances myotube formation in the absence of macrophages. Toward clinical translation, we envision that ATRA-PLG will be injected in the vicinity of debilitated muscle where it can be taken up by macrophages and induce IGF-1 release over a predetermined therapeutic window. Along these lines, we demonstrate that ATRA-PLG microparticles are readily taken up by bone marrow-derived macrophages and reside within the cytosol for at least 12 days with no toxicity. Interestingly, ATRA-PLG induced IGF-1 secretion by thioglycolate-elicited macrophages, but not bone marrow derived macrophages. We found that the RAR isoforms present in lysate differed between the macrophages studied, which could explain the different IGF-1 responses to ATRA. Given that ATRA-PLG enhances myotube formation directly (through ATRA) and indirectly (through macrophage IGF-1) this study supports the further testing of this promising pharmaceutical using rodent models of muscle regeneration and growth.

## 1. Introduction

Muscle atrophy is a highly regulated phenomenon that occurs secondary to disuse, aging, or illness, leading to a decrease in muscle strength, lower quality of life, and higher mortality (Glass, 2003; Zhang et al., 2007). The current standard for treatment involves dietary intervention and exercise, requiring months to years for recovery depending on severity and ability. Moreover, patients undergoing recovery after atrophy become more susceptible to falls and additional injury, prolonging time to recovery. An effective therapy is clearly needed to aid in the muscle recovery progress, yet no pharmaceutical therapies for muscle recovery after atrophy have been approved.

The muscle recovery process (from atrophy and other muscle injuries) is initiated in muscle stem cells called satellite cells. During maintenance or repair, satellite cells proliferate, differentiate into myocytes, then fuse with existing myofibers (Abmayr and Pavlath, 2012). This process is regulated, in part, by macrophages. These macrophages are immune cells that play a significant role in establishing a local environment that maintains the health of the muscle, including regulating activation of satellite cells and existing muscle fibers, promoting muscle regeneration, and maintaining local muscle homeostasis (Tidball, 2017). One of the main muscle-supportive factors that macrophages produce is insulin-like growth factor-1 (IGF-1) (Tonkin, 2015; Pelosi, 2007; DeVol et al., 1990). IGF-1 is a potent coordinator of skeletal muscle growth, regulating muscle cell proliferation and differentiation, which contribute to muscle mass (Barclay et al., 2019; Tidball and Welc, 2015). It is established that exogenous, local delivery of IGF-1 promotes regeneration following severe muscle damage. However, the delivery of proteins like IGF-1 or Interleukin-4 (a known IGF-1 inducer in macrophages) to muscle is difficult due to their short half-life, off-target effects, and high cost (Aguilar et al., 2019; Tayalia and Mooney, 2009). Researchers have been able to induce endogenous expression of IGF-1 in the skeletal muscle of mice through virus-mediated transfer of IGF-1 several months prior to muscle atrophy (Stevens-Lapsley, 2010; Ye, 2013; Park et al., 2014). While preemptive IGF-1 overexpression was able to accelerate recovery after casting- or hindlimb unloading-induced muscle atrophy, this method would be difficult to translate to the clinic considering the difficulty, cost, and regulatory barriers associated with gene therapy in humans (Colella et al., 2018). We suspect that a small molecule inducer of IGF-1 would overcome these difficulties (Tonkin, 2015; Kunkel, 2011).

To find a small molecule inducer of IGF-1, we utilized publicly available databases curating signaling networks and transcriptomics data. In particular, we focused on the Signaling Pathways Project (SPP) (Ochsner et al., 2019). By cross referencing entries from SPP to gene enhancer region entries in GeneCards (Safran, 2022), we found unpublished data, produced by NIH-funded studies, indicating that small molecules could modulate IGF-1 expression in human and rodent macrophages. The transcriptomics data indicate that AM580, a synthetic retinoid acid receptor α (RARα) agonist, increased IGF-1 expression in human blood monocytes nearly four-fold compared to vehicle control (Szeles and Nagy, 2010). RARs are nuclear receptors on gene promotor and enhancer regions that, when activated by small molecule ligands, turn on transcription of the associated genes. There are three isoforms of RAR (α, β and γ) and these receptors are expressed in macrophages and control gene expression (Nagy et al., 2012). While AM580 does not have a track record of use in humans, another RAR agonist, all-trans retinoic acid (ATRA), is FDA approved for several indications including acne (Baldwin et al., 2021) and leukemia (Pillai, 2014; Bhat-Nakshatri et al., 2013) but is not currently approved for muscle-related disease.

Some issues have limited clinical applications of ATRA. Specifically, a short half-life when administered orally (Ozpolat et al., 2003) or intravenously (Kawakami, 2005), poor solubility in aqueous solution (Szuts and Harosi, 1991), and sensitivity to light and oxidation (Tolleson, 2005). Moreover, chronic and high systemic doses have side effects, such as hypertriglyceridemia and mucocutaneous dryness (David et al., 1988). Thus, what is needed is a method to deliver and release ATRA to the skeletal muscle that extends its presence in the tissue and limits off-target delivery and side effects.

Biodegradable biomaterials have been used as drug delivery carriers for over 70 years and present an attractive method for storing and delivering drugs to the intended site (Park, 2019). The rationale to use biomaterial microparticles as a drug delivery system is to improve stability of the delivered molecule, release the drug over time, and direct localization of the drug to specific tissues within the body. Poly(lactide-co-glycolide) (PLG) is a biomaterial with tunable biodegradability and biocompatibility properties. Further, it is employed in 19 FDA approved extended release formulations (Park, 2019; Schoubben et al., 2019), though none of these release ATRA.

ATRA has been encapsulated in PLG particles for experimental treatments of retinal detachment (Giordano et al., 1993), cancer (Errico et al., 2009), and bacterial infection (O’Connor, 2019). However, an ATRA-PLG pharmaceutic designed to modulate macrophage IGF-1 release for the treatment of muscle atrophy has not been described. In this project, we developed ATRA-PLG microparticles for the encapsulation and extended release of ATRA. We then studied this new pharmaceutic for its impact on macrophage IGF-1 release and C2C12 myotube formation, which is an established in vitro system for developing muscle pharmacotherapies (Rupert et al., 2020).

## 2. Methodology

### 2.1. Materials, Equipment and software

The following were purchased from Sigma (St. Louis, MO): 50:50 poly (D,L-lactide-co-glycolide) (PLG) (Resomer 502, MW 7000–17,000), dichloromethane (DCM), Coumarin-6, Poly (vinyl alcohol) (PVA) (MW 13,000–23,000, 87–89 % hydrolyzed), Phosphatase Inhibitor Cocktail, insulin from bovine pancreas, goat serum, horse serum, and phalloidin, Trizma-Base, Trizma-HCl, Glycine, sodium dodecyl sulfate, ammonium persulfate, sodium chloride and brewer’s thioglycollate medium.

The following were purchased from Thermo Fisher (Hampton, NH): Dimethyl sulfoxide (DMSO), ACK lysing buffer, DAPI, Protease Inhibitor Cocktail and phylmethylsulfonyl fluoride, Pierce BCA Protein Assay Kit, SuperSignal West Pico Chemiluminscent, Pierce RIPA Buffer, Hoescht 33342. Ultrapure water was obtained from a Thermo Scientific Barnstead Nanopure system.

The following were purchased from Cayman Chemical (Ann Arbor, MI): all*-trans*-Retinoic Acid (ATRA) and AM580.

The following were purchased from Corning (Corning, NY): DMEM high glucose medium, DMEM/F12 medium, sodium pyruvate, sodium bicarbonate, penicillin–streptomycin and fetal bovine serum, Dulbecco’s phosphate buffered saline, and Trypsin with 0.25 % EDTA.

Red Blood Cell Lysis Solution was purchased from Miltenyi Biotec (Bergisch Gladbach, North Rhine-Westphalia, Germany).

The following were purchased from Biorad (Hercules, CA): Acrylamide/Bis Solution, Laemmli Sample Buffer, Goat anti-mouse IgG (H + L)-HRP Conjugate, mini-PROTEAN Tetra Handcast Systems and Vertical Electrophoresis Cell, Critereon TransBlot, Precision Protein StrepTactin-HRP Conjugate, Precision Plus Protein Dual Color Standards, Goat anti-mouse-HRP Conjugate (STAR207P).

The following were purchased from Cell Guidance Systems (Cambridge, UK): recombinant macrophage stimulating factor (GFM8–10) and recombinant mouse IL-4 (GFM18–20).

The following were purchased from R&D Systems (Minneapolis, MN): IGF-1 DuoSet ELISA kits (DY791), IL-4 DuoSet ELISA (DY404), TNF DuoSet ELISA (DY410), IL-10 DuoSet ELISA (DY417), Myosin Heavy Chain Monoclonal Mouse IgG2B Antibody (MAB4470), Mouse IGF-1 Antibody (AF791) and Normal Goat IgG Control (AB-108-C).

The following antibody was purchased from Invitrogen (Waltham, MA): F(ab’)2-Goat anti-Mouse IgG (H + L) cross-adsorbed secondary antibody conjugated to Alexa Fluor 555 (A-21425).

The following antibodies were purchased from Biolegend (San Diego, CA): TruStain FcX (101319), anti-CD45 clone 30-F11 FITC (103108), anti-F4/80 clone BM8 APC (123116), anti CD11b clone M1/70 PE-Cy7 (101216), Isotype Control Rat IgG2b Clone RTK4530 (400605), Isotype Control Rat IgG2b Clone RTK4530 PE/Cy7 (400618), Isotype Control Rat IgG2a Clone RTK2758 APC (400512).

The following antibody was purchased from Abcam (Cambridge, UK): Goat Anti-Rabbit IgG H&L (ab97051).

The following antibody was purchased from Cell Signaling Technologies (Danvers, MA): RARα Rabbit mAb clone E6Z6K (62294).

The following antibodies were purchased from Santa Cruz Biotechnology (Santa Cruz, CA): mouse anti-RARα clone C-1 (sc-515796), mouse anti-RARβ2 clone B-12 (sc-514585) and mouse anti-RARγ clone G-1 (sc-7387).

The PT3100D homogenizer was purchased from Kinematica (Malters, Switzerland). An Eppendorf Thermomixer R was purchased from Eppendorf (Hamburg, Germany). A Spectramax 190 UV–Vis spectrophotometer was purchased from Molecular Devices (San Jose, CA). A Labconco freeze drier was purchased from Labconco (Kansas City, MO). EVOS FL light microscope with light cubes GFP (470 nm excitation/510 nm emission wavelength), DAPI (357 nm excitation/447 nm emission wavelength) and RFP (530 nm excitation/590 nm emission wavelength) were purchased from Fisher (Hampton, NH). An iBright CL1500 Imaging System was purchased from Thermo Fisher (Hampton, NH). A FACs Area flow cytometer was purchased from BD Biosciences (San Jose, CA).

Software used includes ImageJ as developed and provided by the National institutes of Health and Laboratory for Optical and Computational Instrumentation (Bethesda, MD and LOCI, University of Wisconsin, WN), FlowJo from Becton, Dickinson & Company (Franklin Lakes, NJ), and GraphPad Prism software (San Diego, CA). iBright Analysis Software was provided by Thermo Fisher Scientific (Hampton, NH).

### 2.2. Polymer particle fabrication

PLG particles were made using a single oil-in-water emulsification/solvent evaporation protocol as follows. For the organic phase, PLG and ATRA were dissolved in DCM at concentrations of 52.8 mg/mL and 5 mg/mL, respectively. When fluorescent particles (C6-PLG) were made, coumarin 6 was also added to the DCM at a concentration of 0.5 mg/mL. For the emulsion, 0.6 mL of the organic phase was added dropwise to 4 mL of a PVA solution (10 mg/mL) and homogenized at 11,000 rpm for 5 min. The emulsion was then added to 80 mL of water and stirred at 80 rpm for 1 h, allowing the DCM to evaporate and polymer particles to form. The particles were then passed through a 40 μm filter, collected via centrifugation at 250 ×g for 10 min, and washed 3 times in ultrapure water. Washed particles were frozen at −20 °C and subsequently lyophilized overnight. Recovered particles were stored under vacuum in a dark, dry environment at room temperature.

### 2.3. Particle size measurements

Particle size was determined by analyzing light microscopy images using ImageJ software. Particles were suspended in DMEM at a concentration of 0.25 mg/mL and 400 μL was added to a well of a 48-well plate and allowed to settle prior to image acquisition. Images for particle size analysis were taken on an EVOS FL light microscope at 20X. Three representative images were taken of each particle condition and converted to binary (B/W). The Particle Analysis plugin in ImageJ was used to measure particle diameter. This method was validated with monodispersed polystyrene beads purchased from Duke Standards (Isely et al., 2019).

### 2.4. Quantification of mass yield and drug encapsulation

Mass yield was calculated by Eq. (1), where $M_{tot}$ is the total mass of particles recovered from the emulsion, $M_{PLG}$ is the mass of polymer added to the emulsion, and $M_{RA}$ is the mass of ATRA added to the emulsion.


(1) $$Mass\;\mathrm{Yield}\;(\%)=\left(\frac{M_{tot}}{M_{PLG}+M_{RA}}\right)*100$$

ATRA loading in the particles was determined by dissolving 1 mg of particles in 1 mL DMSO and measuring absorbance at 355 nm using a UV–Vis spectrophotometer. ATRA concentration was then interpolated from a 10-point standard curve generated with standard solutions of ATRA dissolved in DMSO. ATRA loading was calculated by Eq. (2), where $m_{ra}$ is the mass of ATRA measured in a sample of particles weighing $m_{p}$.


(2) $$Drug\;Loading\left(\frac{\mu g}{mg}\right)=\frac{m_{ra}}{m_{p}}$$

Encapsulation Efficiency is calculated by multiplying *Drug Loading* (Eq. (2) by the total mass of particles recovered ($M_{tot}$) and dividing by the mass of ATRA ($M_{RA}$) added to the emulsion as indicated by Eq. (3).


(3) $$\mathrm{Encapsulation}\;\mathrm{Efficiency}\;(\%)=\frac{(\mathrm{Drug}\;\mathrm{Loading})*M_{\mathrm{tot}}}{M_{RA}}*100$$

### 2.5. In vitro release assay

A pre-weighed mass of ATRA-PLG was dispersed in 1 mL ultrapure water in 1.5 mL tubes and maintained at 37 °C and agitated at 600 rpm on an Eppendorf Thermomixer R while protected from light. At the designated time points, tubes were removed from the thermomixer and particles were collected via centrifugation. The supernatant was removed, and the particle pellet was frozen and lyophilized. Particles were analyzed for ATRA loading as described in Section 2.4. ATRA released (%) was calculated by Eq. (4), where $L_{0}$ is the loading of the particles at time point t=0, and $L_{t}$ is the loading at time t during the release assay.


(4) $$\mathrm{Drug}\;\mathrm{Released}\;(\%)=\left(\frac{L_{0}\;-\;L_{t}}{L_{0}}\right)*100$$

### 2.6. Culture of RAW 264.7 macrophages

RAW264.7 (RAW) macrophages were obtained from ATCC and cultured as instructed by the company. RAW macrophages were cultured in DMEM high glucose medium containing 10 % fetal bovine serum, 1 % penicillin–streptomycin, and supplemented with sodium bicarbonate (1.5 % w/v) and sodium pyruvate (1 mM). Cells were seeded at a density of 25,000 cells/cm^2^. Cells were passaged before reaching 60–70 % confluency.

### 2.7. Retinoid treatment of RAW macrophages

Twenty-four hours after seeding, RAW cells were treated with 0.1–10 μM of ATRA or AM580 using DMSO as the vehicle (<0.1 % v/v). After 24 h of treatment, media was collected for ELISA analysis.

### 2.8. ELISA analysis of media

Cell culture media was analyzed with Duoset ELISA kits following the manufacturer instructions.

### 2.9. Particle treatment of RAW macrophages

ATRA-PLG or PLG particles were suspended in RAW culture media (see Section 2.6) and added to wells for final concentrations of 0.5–10 particles per cell seeded. Particle number was calculated assuming spherical geometry with a 2.7 μm diameter and estimating the density of PLG to be 1.6 g/mL (Blasi et al., 2005). After 24 h of treatment, media was collected, and particles were removed by centrifugation.

### 2.10. Cell density measurements

Cell density measurements were made by counting all cells in 500×500 pixel brightfield image taken at 10X. Five images per condition were analyzed and averaged.

### 2.11. Production of conditioned media

RAW macrophage conditioned media (designated as “ii” in Fig. 4): RAW macrophages were seeded in T75 flasks and cultured for 24 h. To generate conditioned media, cells were washed with PBS and 10 mL of fresh media was added to the flasks and placed in the incubator. After 24 h, the media was collected, filtered through a 0.2 μm filter, and frozen in aliquots at −20 °C.

ATRA-PLG conditioned media (designated as “iii” in Fig. 4): 10 mL of fresh media containing 100 μg of ATRA-PLG particles were added to T75 flasks and placed in the incubator. After 24 h, the media was collected, filtered through a 0.2 μm filter, and frozen in aliquots at −20 °C.

RAW macrophage treated with ATRA-PLG conditioned media (designated as “iv” in Fig. 4): RAW macrophages were plated in T75 flasks and cultured for 24 h. To generate conditioned media, cells were washed with PBS and 10 mL of fresh media containing 100 μg of ATRA-PLG particles were added to flasks and placed in the incubator (100 μg corresponds to 10 particles per cell seeded). After 24 h, the media was collected, sterile-filtered through a 0.2 μm filter, and frozen in aliquots at −20 °C.

### 2.12. C2C12 myoblast culture and myotube formation assay

C2C12 myoblasts were obtained from ATCC and cultured as instructed by the company. C2C12 cells were cultured in DMEM high glucose medium supplemented with 10 % fetal bovine serum and 1 % penicillin–streptomycin. Cells were seeded at a density of 25,000 cells/cm^2^. Media was changed every other day and cells were passaged before reaching 70 % confluency.

To induce myotube formation, C2C12 cells were cultured to confluence, at which time the media was changed to “Differentiation Media” consisting of DMEM high glucose medium supplemented with 1 μM bovine insulin, 2 % horse serum, and 1 % penicillin–streptomycin for twenty-four hours. For the following four days, the media was exchanged daily with fresh media consisting of a 1:1 ratio of DMEM high glucose medium supplemented 2 % horse serum and 1 % penicillin–streptomycin and one of the following: (i) “unconditioned media”, which was fresh RAW macrophage culture media (section 2.6); (ii) media conditioned with RAW macrophages for twenty-four hours (section 2.11); (iii) media conditioned with ATRA-PLG for twenty-four hours (section 2.11); or (iv) media conditioned with RAW macrophages treated with ATRA-PLG for twenty-four hours (section 2.11). After three consecutive days of exposure to treatment media, myotube cultures were fixed in 4 % formaldehyde. In certain experiments, an IGF-1-neutralizing antibody or Goat IgG (isotype control) was added to the treatment media (i.e., i, ii, iii, or iv) at 1 μg/mL.

### 2.13. Immunofluorescence microscopy of myotubes

Immunofluorescence was conducted on 4 % paraformaldehyde-fixed myotubes using a mouse anti-myosin heavy chain primary antibody (1 μg/mL) and a goat anti-mouse antibody conjugated to Alexa Fluor 555 (10 μg/mL). Nuclei were visualized with DAPI. Light and fluorescent images of cells were taken on an EVOS FL light microscope at 10X and 20X. Fluorescence images were acquired with the DAPI and RFP light cubes.

### 2.14. Myotube image analysis

C2C12 myotubes were quantified using ImageJ software. The number of nuclei in each myotube was counted and a myotube was defined as a Myosin Heavy Chain-positive tube containing three or more nuclei. For each well, 3–5 random fields were imaged and quantified.

### 2.15. Harvest and culture of bone marrow-derived macrophages (BMDM)

Primary cultures of bone marrow-derived macrophages (BMDM) were generated from the hindlimbs of female ICR (CD-1) mice (Inotiv formally Envigo formally Harlan, Frederick, MD) between 8 and 12 weeks old. After euthanasia, hindlimbs were dissected and marrow was collected through centrifugation. Red blood cells were lysed, and remaining cells were seeded into non-tissue culture-treated petri dishes at 500,000 cells/cm^2^. Cells were cultured in DMEM/F12 media containing 10 % FBS, 1 % Penicillin-Streptomycin (BMDM Complete Media), and supplemented with 10 ng/mL macrophage-colony stimulating factor (M–CSF). After 5 days of differentiation, nonadherent cells were removed and the adherent population was passaged into tissue culture-treated multi-well plates at a seeding density of 50,000 cells/cm^2^. BMDMs seeded into multi-well plates received ½ media changes every 48 h in which ½ of the media was removed and replaced with an equal volume of fresh BMDM Complete Media supplemented with 10 ng/mL M–CSF.

### 2.16. Flow cytometry of BMDMs to assess purity

After 5 days of differentiation, BMDMs were trypsinized and washed with MACS buffer (PBS, 0.5 mM EDTA, 30 % BSA), and incubated with TruStain FcX followed by antibodies to CD45, CD11b, and F4/80. After antibody incubation, cells were washed, fixed, and analyzed using a FACS Aria flow cytometer. FlowJo software was utilized to compensate and analyze data. FMOs with isotype controls were used to determine specific antibody signal. The gating scheme used in the flow cytometry analysis is depicted in Fig. 5A.

### 2.17. Particle and IL-4 treatment of BMDMs

Forty-eight hours after being seeded in multi-well plates, BMDMs were treated with PLG, C6-PLG, or ATRA-PLG at a concentration of 1, 5, 10, or 50 particles per cell seeded. After 48 h of particle treatment, the media was collected and depleted of particles by centrifugation and particles remaining in the well were removed by gentle washing with PBS. The particle-depleted media was added back to wells with fresh BMDM Complete Media supplemented with 10 ng/mL M–CSF at a 1:1 ratio for a ½ media change. The BMDMs were then cultured with these ½ media changes every 48 h for up to 12 days.

Note, for one experiment, BMDMs were treated with IL-4. After 48 h of IL-4 treatment, a ½ media change was carried out and no additional IL-4 was added to the well. These ½ media changes continued every 48 h for 12 days when the experiment was terminated.

### 2.18. Treatment of BMDMs with ATRA in the absence of M–CSF

Forty-eight hours after BMDMs were seeded into tissue culture-treated multi-well plates (see section 2.15), media was exchanged with fresh BMDM Complete Media without M–CSF. Twenty-four hours later, ATRA (final concentrations: 0.1, 1, or 10 μM) or DMSO (<0.1 % v/v) was added to existing media. Media was then collected after 24 h.

### 2.19. Harvest, culture, and treatment of primary peritoneal macrophages

Primary cultures of peritoneal macrophages were generated from C57BL/6J mice. Mice were injected with 1 mL 3.8 % brewer’s thioglycollate medium into the peritoneal cavity. Three days later, mice were euthanized and a peritoneal lavage with DPBS was used to collect peritoneal macrophages. The aspirated cell suspension was centrifuged at 400 g for 10 min at 4 °C, and the supernatant was discarded. Red blood cells were removed using Red Blood Cell Lysis Solution. The pellet was resuspended, counted, and plated in DMEM + 10 % FBS + 1 % Penicillin/Streptomycin. Twenty-four hours later, ATRA (final concentrations: 0.1, 1, or 10 μM) or DMSO (<0.1 % v/v) was added to existing media. Media was then collected after 24 h.

### 2.20. Western Blot

Cell pellets were generated from untreated RAW, BMDMs, or peritoneal macrophages. The cell pellet was lysed in RIPA buffer containing phosphatase inhibitors, protease inhibitors, and 1 mM phenylmethylsulfonyl fluoride, and centrifuged to generate whole cell lysate. The protein content of the lysate was measured using BCA protein assay kits. For Western blotting, 30 μg of protein was separated by SDS-PAGE on 10 % polyacrylamide gels. The bands were transferred to nitrocellulose membranes, and then Ponceau S staining and imaging was performed. The membrane was then blocked with 5 % nonfat milk and incubated overnight at 4 °C with one of the following antibodies: anti-RARα (1:250), anti-RARβ (1:1000), or anti-RARγ (1:250). Afterwards, membranes were rinsed and incubated with goat anti-mouse or goat anti-rabbit secondary antibody (1:1000) diluted in 5 % nonfat milk for an hour. Bands were visualized using chemiluminescence substrate. Images of the membranes were acquired, scanned, and analyzed using iBright Analysis Software.

### 2.21. Statistical analysis

GraphPad Prism software was employed for all statistical analysis. Where appropriate, a *t*-test, one-way or two-way ANOVA test with Tukey’s multiple comparison were carried out to compare differences between means. Data represented as mean ± standard deviation unless otherwise stated. Statistical symbols indicate *p-values* of * < 0.05, ** < 0.01, *** < 0.001, and **** <0.0001 to show significant difference between the groups unless noted otherwise. Additional details regarding statistical analyses carried out for each data set are described in the figure legend.

## 3. Results

### 3.1. Induction of IGF-1 secretion in RAW macrophages with ATRA

**Fig. 1.**
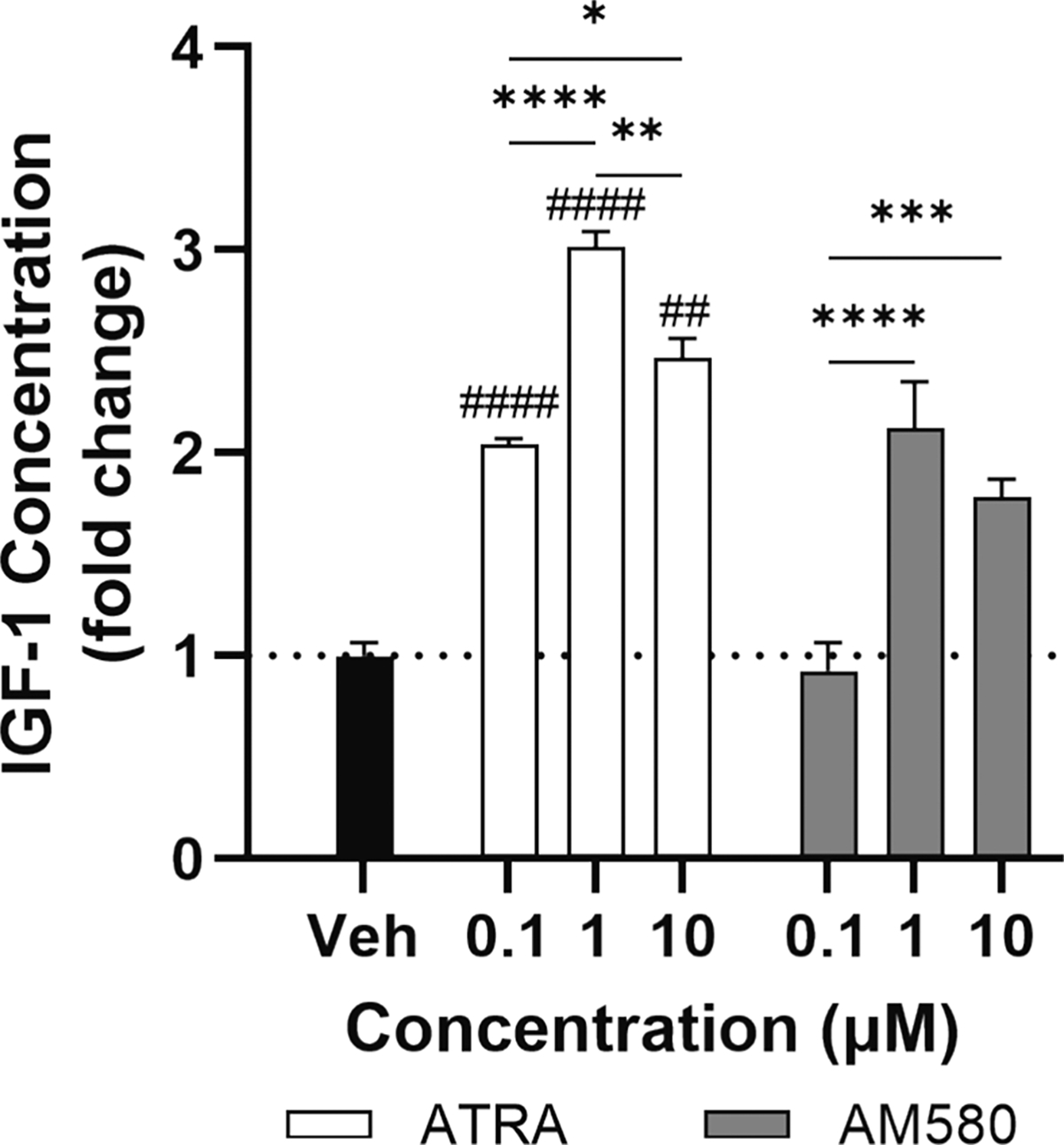
Impact of ATRA and AM580 on IGF-1 secretion by RAW macrophages. RAW macrophages were treated with 0.1, 1, or 10 μM of ATRA or AM580. A DMSO vehicle control (Veh) was also included. After 24 h, media was collected and IGF-1 was measured with ELISA. Two-way ANOVA with Tukey’s multiple comparison test was conducted with drug and dosage as sources of variation. ## and #### indicates p < 0.01 and p < 0.0001, respectively, compared to equivalent AM580 dose. *, **, ***, and **** indicates p < 0.05, 0.01, 0.001, and 0.0001, respectively. Data is representative of three independent experiments with three technical replicates per treatment condition. Data are expressed as mean ± SD.

IGF-1 is a key factor in muscle regeneration and growth. Macrophages are sources of IGF-1 in the muscle (Tidball, 2017) and unpublished transcriptomics dataset available through the Signaling Pathways Project website (Szeles and Nagy, 2010) indicated that activation of retinoic acid receptors in macrophages induces IGF-1 gene expression. Thus, we tested the ability of retinoids AM580 and ATRA to induce IGF-1 secretion into the culture media using RAW macrophages (Fig. 1). IGF-1 concentration for the vehicle control (DMSO) was 20 ± 1 pg/mL, and all other measurements are normalized to this value. All tested ATRA concentrations increased IGF-1 expression with the largest increase (3-fold) achieved at 1 μM. In the same experiments (i.e., on the same well plates), we also investigated the effect of AM580. We found that only 1 and 10 μM AM580 increased IGF-1 over vehicle control. In addition, at every concentration tested, ATRA induced significantly more IGF-1 than AM580.

### 3.2. Characterization of ATRA-PLG particles

**Fig. 2.**
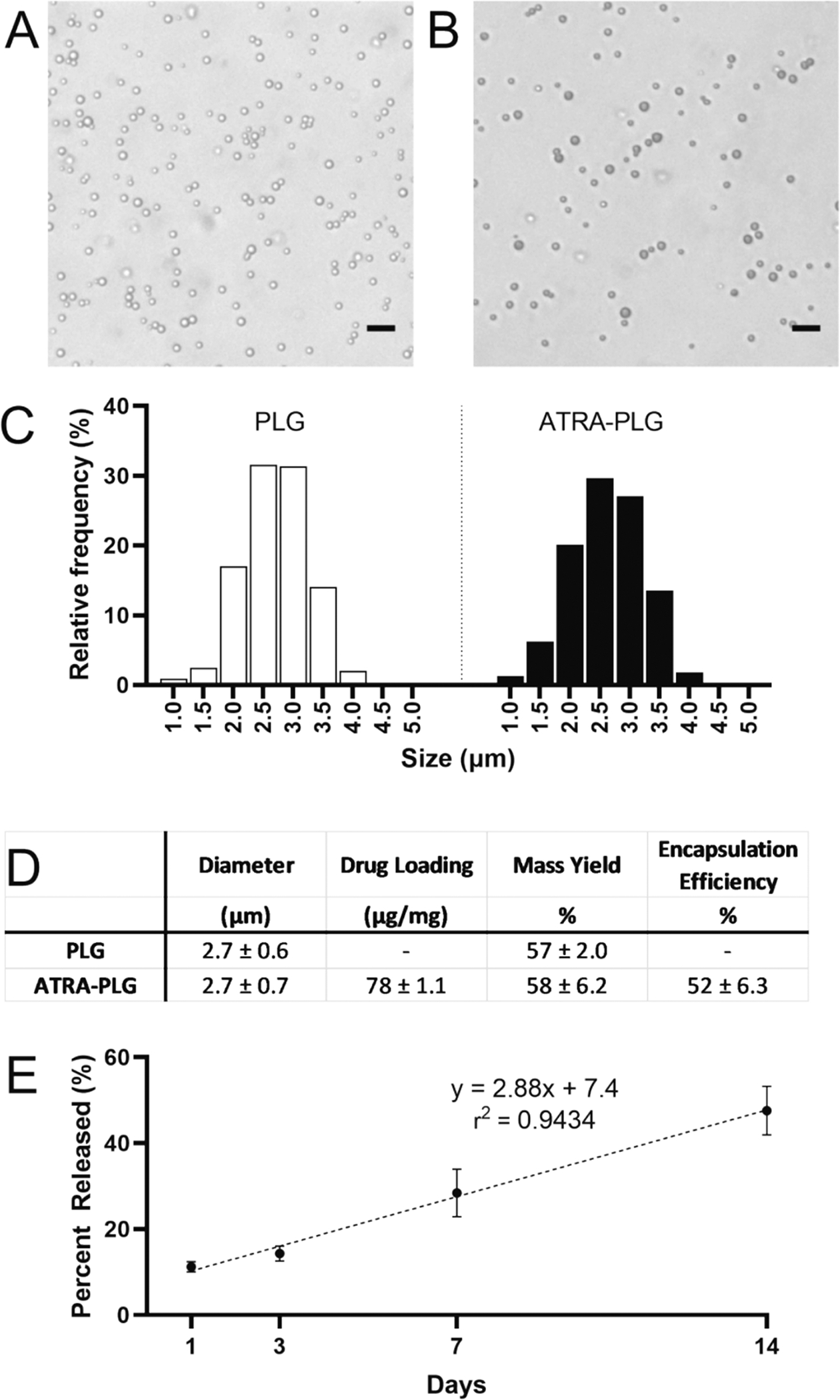
Characterization of ATRA-PLG particles. Images of (A) PLG and (B) ATRA-PLG microparticles. Scale bar = 5 μm. (C) Histograms of particle size and (D) characterization of drug loading and size of particles. Data is representative of three independent particle fabrications, in which three beakers of each particle type were produced. (E) ATRA drug release profile from particles over 14 days. Linear regression indicated by the dashed line. Data is representative of two independent experiments with three technical replicates per time point. Data is mean ± SD.

Two particle formulations were produced: PLG particles with no drug loading (PLG) or PLG particles loaded with ATRA (ATRA-PLG). All particles were spherical (Fig. 2A–B) with a narrow size range (Fig. 2C). The ATRA-PLG particles were yellow in color and appeared darker via microscopy compared to the PLG particles, which were white (Fig. 2A–B). Particle characteristics are listed in Fig. 2D. The average particle diameter was 2.7 μm and mass yield was approximately 57 %. The addition of ATRA to the emulsion did not impact average particle size or mass yield under the conditions tested. Drug loading and encapsulation efficiency for ATRA-PLG was 78 μg/mg and 52 %, respectively.

Having determined that ATRA was encapsulated into PLG particles, we next investigated the release kinetics of ATRA from the particles in vitro (Fig. 2E). ATRA-PLG particles exhibited a linear drug release of 2.9 % per day (r^2^ = 0.943) between days 1 and 14.

### 3.3. Impact of ATRA-PLG on RAW macrophage IGF-1 secretion

**Fig. 3.**
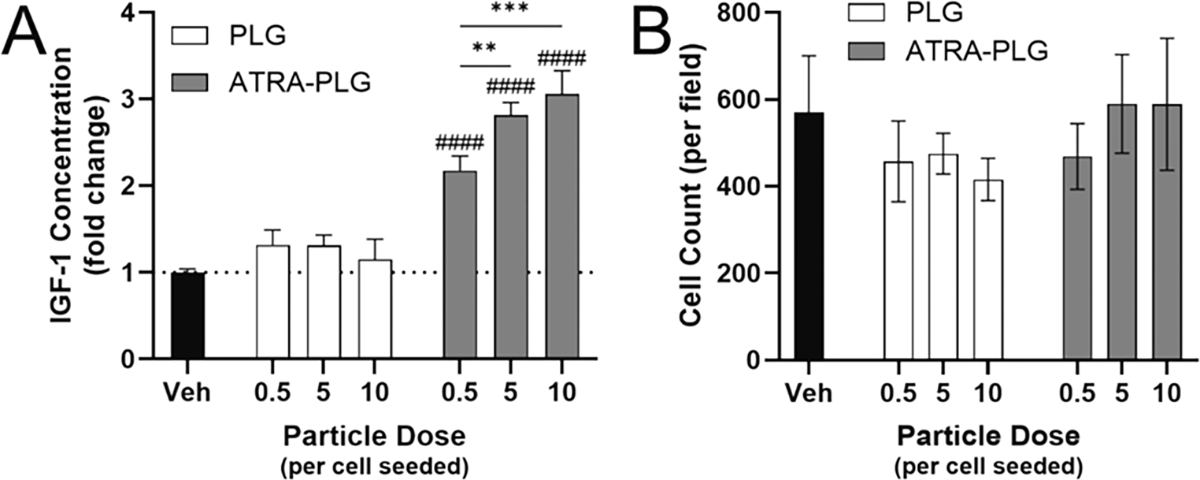
24-hour treatment of RAW macrophages with ATRA-PLG particles. RAW macrophages were treated for 24 h with PLG or ATRA-PLG particles at 0.5, 5, or 10 particles per cell seeded, or with a vehicle control (Veh, media). (A) IGF-1 in the media measured by ELISA and normalized to vehicle control, which was 21 ± 1 pg/mL. (B) Cell density measurements. Two-way ANOVA with Tukey’s multiple comparison was conducted between PLG and ATRA-PLG with particle type and dose as sources of variation. #### indicates p < 0.0001 versus PLG particles at the equivalent dose. ** and *** indicate p < 0.01 and 0.001, respectively, between the groups as indicated. Data is representative of 5 independent experiments each conducted with three technical replicates per condition. Data is mean ± SD.

Having developed ATRA-PLG particles, we next investigated if the ATRA remained able to induce IGF-1 expression after encapsulation. To test this, we exposed RAW macrophages to a dose range of particles for 24 h and then collected the media. The doses investigated were 0.5, 5 and 10 particles per cell seeded. ATRA-PLG particles significantly increased IGF-1 expression over blank particles (Fig. 3A), indicating the ATRA released was bioactive. A dose response was detected for the ATRA-PLG particles, with no difference detected between the two highest doses. Microscopic analysis of cell morphology suggested that the amount of ATRA released was well tolerated by the cells and cell density measurements made from those images was not impacted (Fig. 3B). Also, we assessed the long-term stability of ATRA-PLG. We found that particles stored at room temperature under vacuum and in the dark for 3 months induce an IGF-1 response comparable to particles stored for only 1 week (Supplementary Figure 1). The data indicate an ATRA-PLG shelf life of at least 3 months when stored under dry conditions protected from light.

### 3.4. C2C12 myotube formation from conditioned media collected from ATRA-PLG treated RAW macrophages

**Fig. 4.**
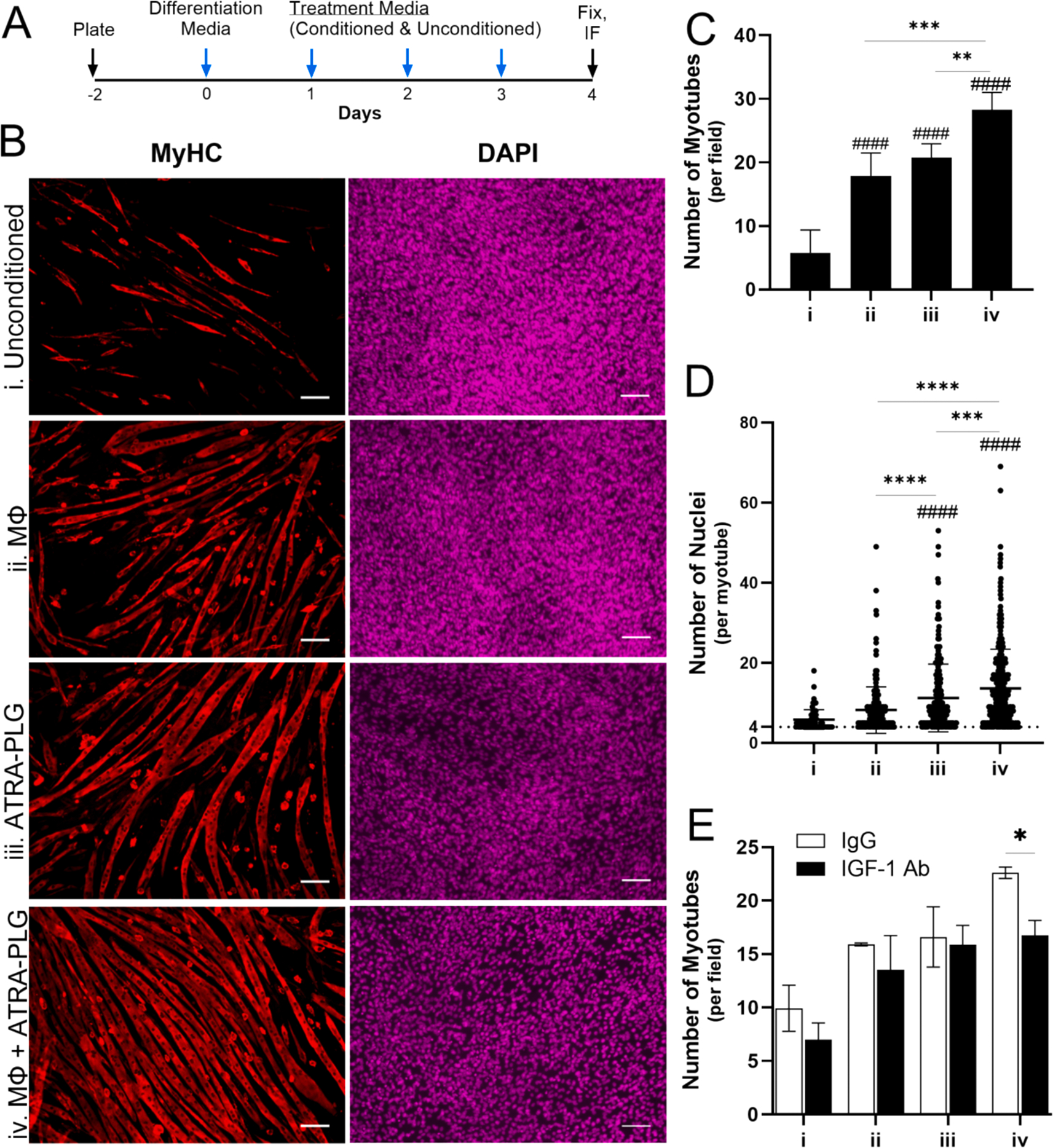
Impact of conditioned media on C2C12 myoblast formation. (A) Timeline of experiment. Blue arrows indicate media changes. (B) Representative immunofluorescence images of myotubes after conditioned media treatment, probed with a myosin heavy chain (MyHC) antibody and counterstained with DAPI. Scale bar = 100 μm. (C) Number of myotubes per field of view. (D) Number of nuclei per myotube where horizontal lines denote mean and standard deviation. (E) Number of myotubes when using an IGF-1-neutralizing antibody or an IgG control. (C-D) One-way or (E) Two-way ANOVA was conducted between means where #### indicates p < 0.0001 compared to (i) and *, **, ***, and **** indicates p < 0.05, 0.01, 0.001, and 0.0001, respectively, between indicated groups. Data is from three (C and D) or one (E) independent experiments with three technical replicates per treatment condition. Data are expressed as means ± SD. IF = immunofluorescence microscopy. (For interpretation of the references to color in this figure legend, the reader is referred to the web version of this article.)

ATRA-PLG treatment of RAW macrophages induced IGF-1 secretion (Fig. 3A), which is a protein important for satellite cell proliferation and differentiation into mature muscle (Engert et al., 1996). Thus, we sought to study the impact of media from RAW macrophages treated with ATRA-PLG on myotube formation using C2C12 myoblasts. To accomplish this, RAW macrophages were treated with ATRA-PLG particles for 24 h and then the “conditioned” media was collected, depleted of particles via filtration, and stored for later treatment of C2C12 cells (this media is referred to as media “iv”). Also, unconditioned media (i), RAW macrophage conditioned media without particles (ii) and ATRA-PLG conditioned media (iii) were generated as controls. As shown in the timeline in Fig. 4A, after one day of differentiation, the unconditioned (i) or conditioned medias (ii-iv) were used to differentiate myoblasts for 3 days. Then immunofluorescence microscopy was performed for myosin heavy chain and nuclei (Fig. 4B). Differentiation was terminated after 4 days instead of later time points used in previous literature (e.g., 7 days) (Rupert et al., 2020). We did this because the conditioned media caused the C2C12 cells to form myotubes rapidly and by day 5 the mature myotubes detached from the cell culture dish making them difficult to image. We found that media collected from macrophages treated with ATRA-PLG particles (media iv) induced C2C12 cells to fuse more readily into myotubes compared to all other treatments, as quantified by the number of myotubes per image field (Fig. 4C) and the number of nuclei per myotube (Fig. 4D). The data indicate that all conditioned medias enhanced C2C12 myotube formation; however, media from RAW macrophages treated with ATRA-PLG was superior in this respect.

We hypothesized that the superior myotube growth with media from RAW cells treated with ATRA-PLG (media iv) was due to IGF-1. To test this, we conducted the same myotube formation assay with an IGF-1-neutralizing antibody or antibody without IGF-1 specificity. We found that IGF-1 neutralization significantly decreased myotube formation when C2C12 cells were treated with conditioned media from RAW macrophages treated with ATRA-PLG (Fig. 4E, media iv). In contrast, the other groups exhibited a non-significant decrease in myotube formation when IGF-1 was neutralized. The data suggest that increased IGF-1 content is responsible for the increased myotube formation observed using conditioned media from RAW macrophages treated with ATRA-PLG (media iv).

It is important to consider that IGF-1 antibody blockade tends to decrease myotube formation when the C2C12 cells are treated with control medias (particularly medias i and ii, Fig. 4E); although, the differences in means were not statistically significant. These findings suggest that C2C12 cells may also be a source of IGF-1 that impacts myotube formation. Thus, the significant reduction in myotube formation for media iv may be due to antibody blockade antagonizing IGF-1 growth signals from both C2C12 cells and macrophages.

### 3.5. Long-term culture of bone marrow derived macrophages with florescent particles

**Fig. 5.**
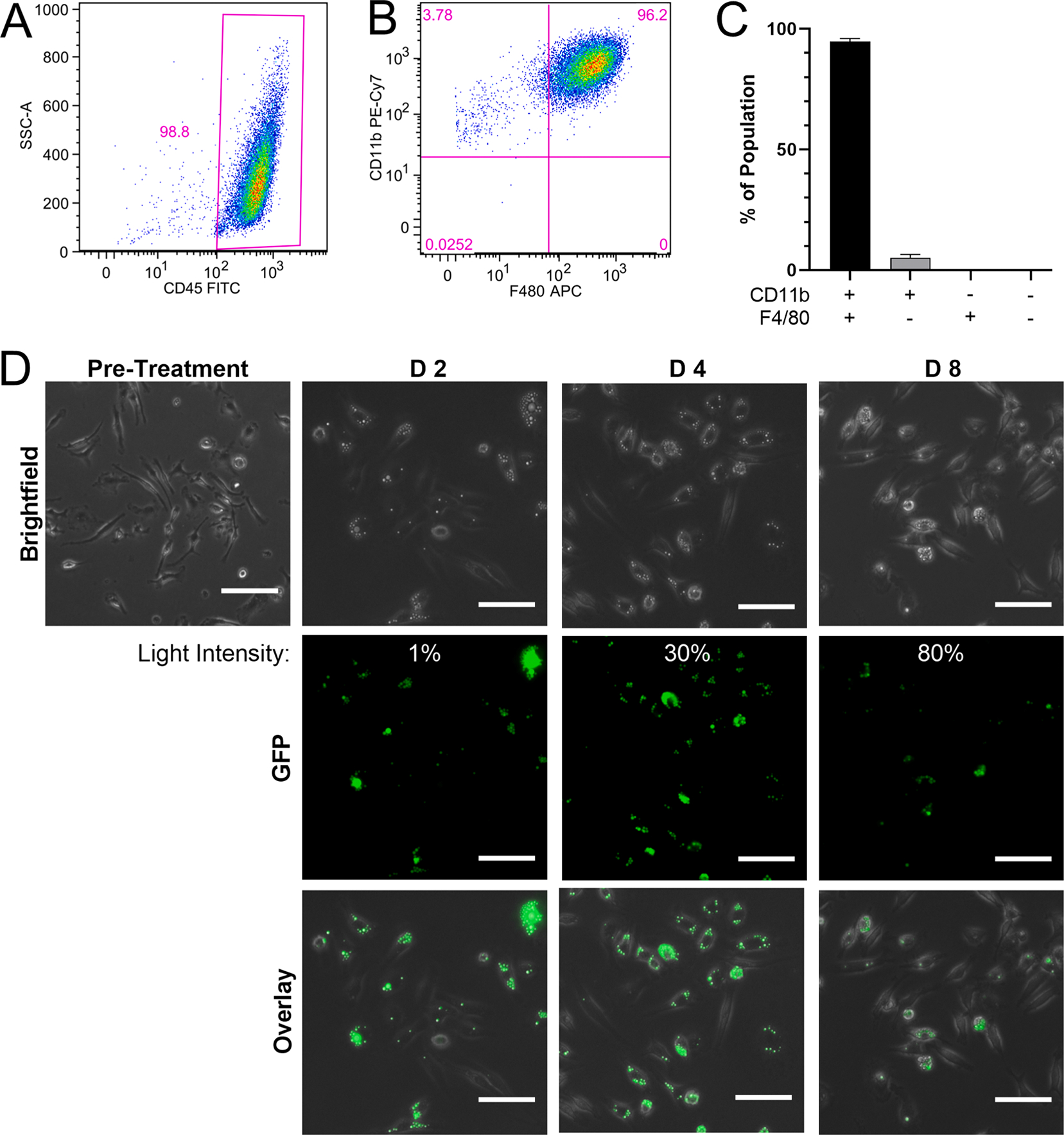
Culture and treatment of BMDMs with fluorescent Coumarin-6 particles. Representative flow cytometry graphs of (A) Side scatter vs. CD45 and (B) CD11b vs F4/80 cells 4 days after harvest and differentiation. (C) Proportion of CD11b + and F4/80 + cells among total cell population. (D) Live images of fluorescent C6-PLG particles (GFP filter cube) in BMDMs over 8 days in culture. Scale bar = 50 μm. Images are representative of three independent experiments.

The eventual application of ATRA-PLG particles will be their delivery to muscle tissue where they may be taken up by macrophages, reside in the cytosol, and release ATRA for an extended period. Thus, we were interested in determining the fate of particles cultured with terminally differentiated bone marrow derived macrophages (BMDM), which do not divide and can be cultured for days without passage (in contrast to RAW macrophages). To aid in visualization, the particles encapsulated coumarin 6 (C6), a fluorescent dye with comparable molecular weight and hydrophobicity to ATRA (PubChem, 2022; PubChem, 2022). Importantly, bone marrow cell differentiation consistently yielded cultures in which greater than 95 % of the cells expressed F4/80 and CD11b (Fig. 5 A–C), which are accepted markers for BMDMs (Davies and Gordon, 2005). In addition, BMDMs could be cultured without passage for up to 21 days with ½ media changes every two days (data not shown). With these tools in place, BMDM were treated with C6-PLG particles (size = 2.7 μm) at a dose of 50 particles per cell seeded. Two days later the unbound/non-internalized particles were removed during a ½ media change and then the cells were imaged using fluorescence microscopy (Fig. 5D). The ½ media changes and fluorescence imaging continued every two days until day 8. We found that after 8 days in culture, fluorescent C6-PLG particles were still intact; however, particle fluorescence waned over time and required higher excitation light intensity for detection (Fig. 5D). This was likely due to the release of the coumarin 6 overtime.

### 3.6. Impact of ATRA-PLG on IGF-1 secretion from bone marrow derived macrophages

**Fig. 6.**
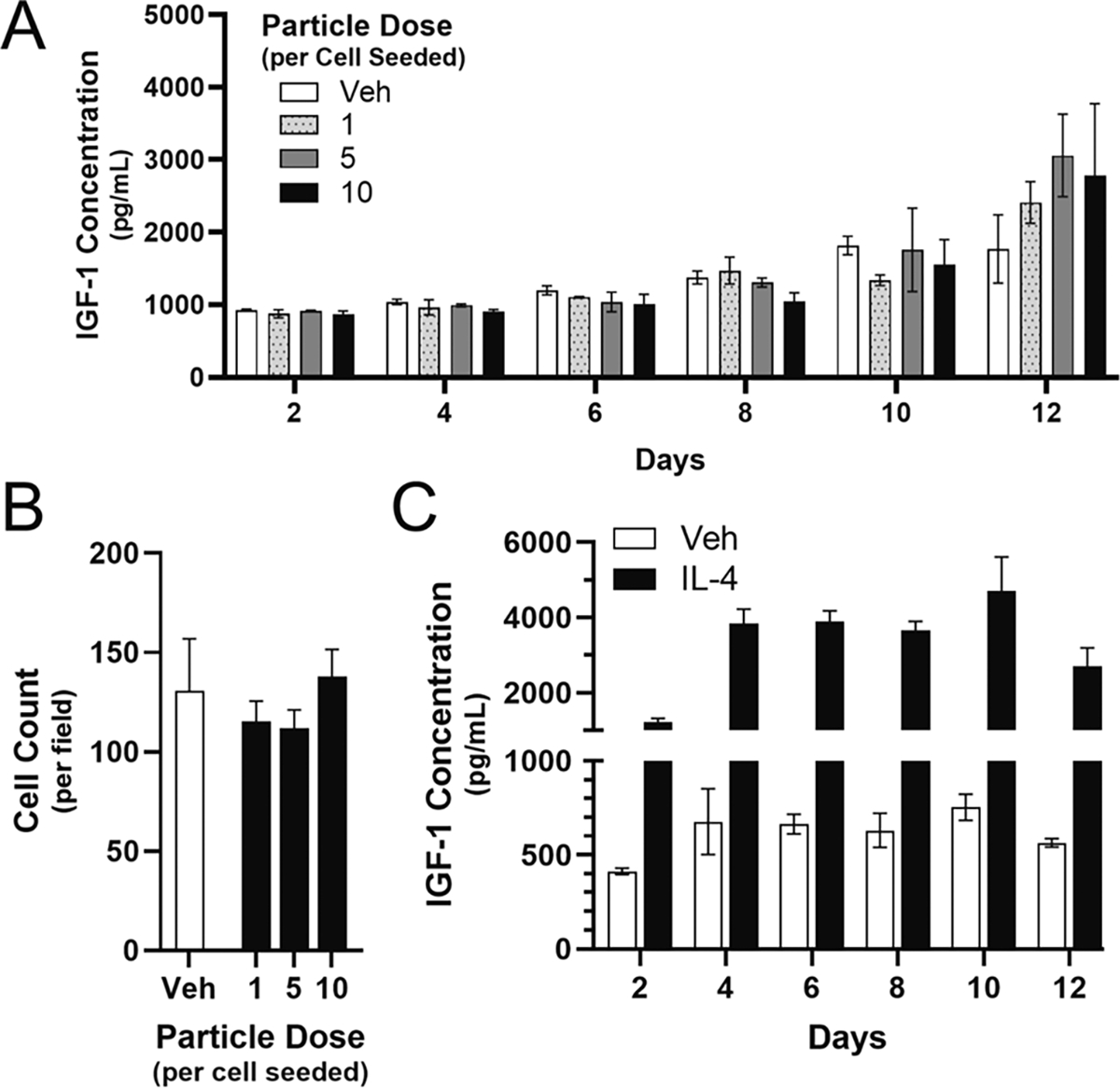
Culture and treatment of BMDMs with ATRA-PLG particles. (A) IGF-1 content in media during 12 days of culturing ATRA-PLG with BMDMs. (B) Cell density measurements after 12 days. (C) IGF-1 concentration over 12 days after treating BMDMs with 5 ng/mL of IL-4 on day 0. Two-way ANOVA (A) or One-way ANOVA (B) was conducted. Data is representative of two (A-B) or one (C) independent experiments with three technical replicates per condition at each time point. Data are expressed as means ± SD.

We next investigated the impact of ATRA-PLG particles on secretion of IGF-1 from BMDM cultures over time. To accomplish this, we treated BMDM with a dose range of ATRA-PLG particles in the same manner as Fig. 5. Briefly, cells were treated with particles for 48 h and then unbound particles were removed during a ½ media change. Then, every 2 days for 12 days, ½ media changes were carried out while ½ of the conditioned media was frozen. At day 12 we confirmed that particles were visible in the ATRA-PLG treated BMDM and then terminated the experiment (Supplementary Figure 2). We then conducted an IGF-1 ELISA on the media from all time points. Surprisingly, we did not detect changes in IGF-1 levels in comparison to Vehicle-treated cells at any concentration between days 2 and 10. However, at day 12, there was a trend of increased IGF-1 for the ATRA-PLG treated cells (Fig. 6A). Analysis of images taken on day 12 demonstrated that cell density was not impacted over the course of the experiment (Fig. 6B) indicating that a decreased cell count did not play a role in the IGF-1 response. We also confirmed that the BMDMs could increase expression of IGF-1 in response to IL-4 treatment (Fig. 6C) ruling out the possibility that BMDMs in our studies could not increase IGF-1 production. IL-4 treated BMDMs were able to maintain high levels of IGF-1 for up to 12 days with a single, two-day treatment of IL-4, which was followed by ½ media changes every 2 days with fresh media that did not contain IL-4 (Fig. 6C, black bars).

### 3.7. Investigating conditions in which ATRA induces IGF-1 production by primary macrophages

**Fig. 7.**
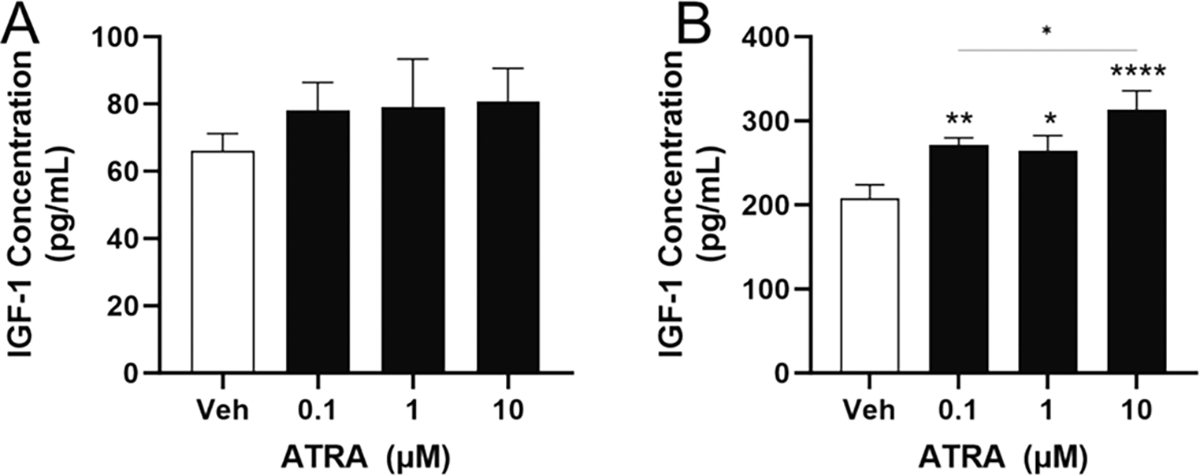
IGF-1 expression in primary macrophages without M¡CSF in the culture. (A) IGF-1 in media after 24 h of treating BMDMs with ATRA in the absence of M–CSF. (B) IGF-1 in media after 24 h of treating peritoneal macrophages with ATRA, which do not require M–CSF. One-way ANOVA was conducted where *, **, ***, and **** indicates p < 0.05, p < 0.01, p < 0.001, and p < 0.0001 compared to vehicle, respectively, unless otherwise stated. Data is from one (B) or two (A) independent experiments with three technical replicates per condition. Data is mean ± SD.

Our data indicated that either ATRA or ATRA-PLG induces IGF-1 secretion by RAW macrophages (Figs. 1 and 3A), and we were surprised when a similar IGF-1 response was not observed with BMDMs (Fig. 6A). Literature indicates that M–CSF, a growth factor needed for the differentiation of BMDMs, induces IGF-1 expression in these cells (Arkins et al., 1995). Thus, we questioned if M–CSF was interfering with our ability to detect IGF-1 production in response to ATRA. We determined that once the BMDMs were fully differentiated, M–CSF could be removed from the culture for up to 72 h before viability was affected (data not shown). Having established this, we then treated BMDMs, which had not been exposed to M–CSF for 24 h, with ATRA; however, BMDMs did not exhibit a significant increase in IGF-1 content in media (Fig. 7A). To determine if ATRA can induce IGF-1 production in other primary macrophages, peritoneal macrophages (which do not require M–CSF for culture) were cultured in the presence of ATRA for 24 h. Interestingly, ATRA did induce IGF-1 in peritoneal macrophages (Fig. 7B).

**Fig. 8.**
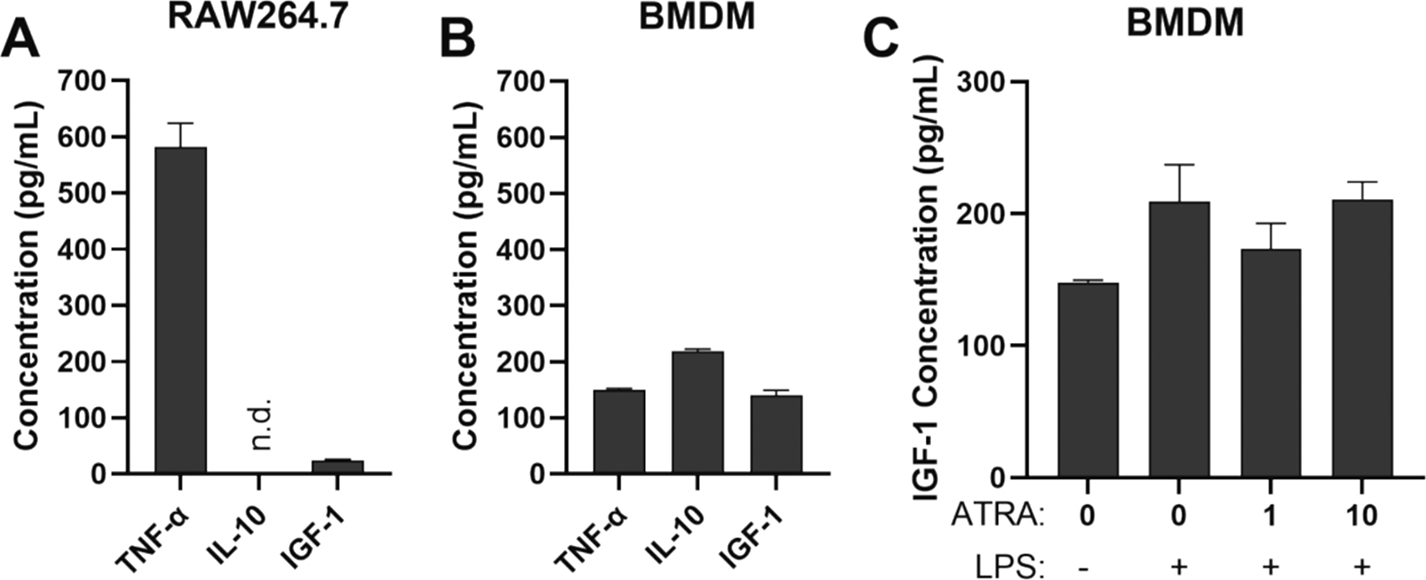
Cytokines produced by macrophages during culture and impact of LPS on IGF-1 release by BMDM treated with ATRA. TNF-α, IL-10 and IGF-1 levels in culture media of (A) RAW macrophages and (B) BMDM. Cytokines were measured by ELISA after 24-hours of culture. n.d. means not detected. (C) IGF-1 levels in culture media from BMDMs treated with LPS (1 ng/mL) for 6 h and then treated with ATRA (1 or 10 ng/mL) for 66 h. IGF-1 measured by ELISA. Data is from 2 independent experiments with 3 technical replicates per experiment. Data are mean ± SD.

We investigated whether differences in activation states between RAW macrophages and BMDMs could explain the variations in IGF-1 production observed. To determine the activation state of resting (i.e., unstimulated) RAW macrophages and BMDM, we conducted ELISA for TNF-α, IL-10, and IGF-1 in culture media. We found that RAW macrophages express high levels of TNF-α and low levels of IL-10 and IGF-1 compared to BMDM (Fig. 8A and B). The data indicate that RAW macrophages exhibit a pro-inflammatory activation state compared to BMDM. This finding led us to investigate if inflammatory activation of BMDM with LPS could enhance IGF-1 release after ATRA treatment. However, this was not the case. While LPS treatment tended to increase IGF-1 release by BMDM, ATRA treatment following LPS treatment did not further increase IGF-1 (Fig. 8C). Taken together, the data indicate that RAW macrophages exhibit a more pro-inflammatory activation state compared to BMDM in terms of the cytokines measured. However, inflammatory activation of BMDM with LPS is not sufficient to increase IGF-1 release after ATRA treatment.

### 3.8. Investigating retinoic acid receptor in macrophage types

**Fig. 9.**
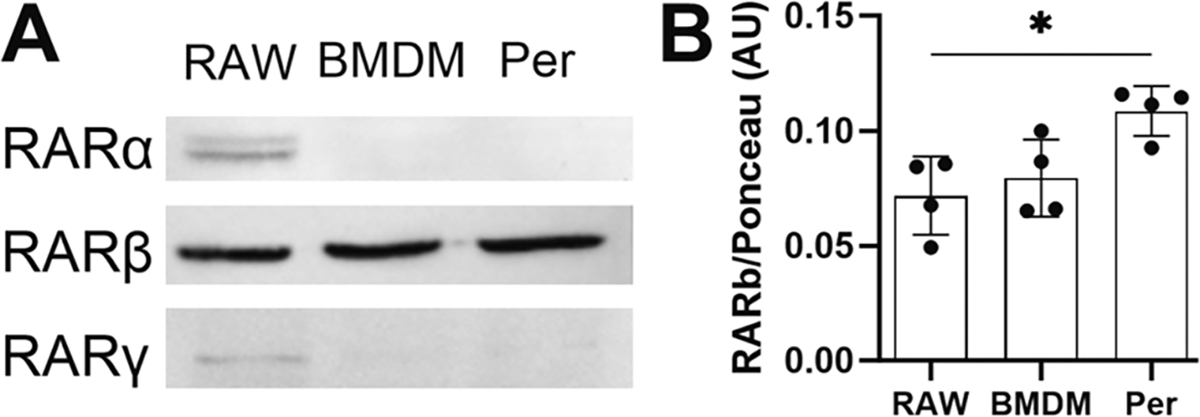
RAR expression in macrophages. (A) Representative western blots of RARα (antibody clone E6Z6K), RARβ (antibody clone B-12), and RARγ (antibody clone G-1) in RAW, BMDMs and peritoneal (Per) macrophages. (B) Intensity of RARβ signal divided by relative ponceau stain intensity. One-way ANOVA with Tukey’s was conducted where * indicates p < 0.05. n = 4 independent experiments. Data are expressed as means ± SD.

The differing IGF-1 response (to ATRA) in RAW, BMDMs, and peritoneal macrophages suggested that there may be a difference in expression of retinoic acid receptors (RAR) in these cell types. Western blots for RARα, RARβ, and RARγ were conducted on whole cell lysate from RAW cells, BMDMs, and peritoneal macrophages. RARβ was detected in all three types of macrophages; in contrast, RARα and RARγ were only detected in RAW macrophages (Fig. 9A). Equivalent protein loading was confirmed with Ponceau-S stain (Supplementary Figure 3). Also, the absence of RARα protein in BMDMs and its presence in RAW macrophages was confirmed with a second antibody clone (Supplementary Figure 4). Finally, we quantified RARβ expression and determined the level in peritoneal macrophages was significantly higher than in RAW macrophages (Fig. 9B).

## 4. Discussion

We present here, for the first time, that RAR agonists modulate IGF-1 secretion in several types of macrophages. ATRA and AM580 induced IGF-1 expression in RAW macrophages, and ATRA induced IGF-1 in peritoneal macrophages, but not in BMDMs. We also developed a PLG microparticle delivery system that maintains ATRA bioavailability and released the molecule over a time frame of weeks. In a C2C12 myotube formation assay, media from ATRA-PLG treated RAW macrophages enhanced myotube formation and this effect was inhibited by an IGF-1 neutralizing antibody. Finally, we determined that nuclear receptors for ATRA (i.e., RARα, RARβ, and RARγ) are differentially expressed among RAW, BMDM, and peritoneal macrophages providing a plausible mechanism for the IGF-1 responses observed after ATRA treatment in each cell type. In this section, we discuss these findings.

We found the IGF-1 response to ATRA differed among the macrophage types studied. Specifically, RAW macrophages and peritoneal macrophages produced IGF-1 in response to ATRA but BMDMs did not. While we were surprised by this finding, it is important to consider that macrophages from various tissues differ in gene expression of many factors including, insulin-like growth factor-1, fatty acid binding protein-1, fibroblast growth factor-1, and transforming growth factor-beta (The Immunological Genome Consortium et al., 2012; Berghaus et al., 2010). Also, we are not the first to report phenotypic differences between RAW cells and BMDMs. For instance, RAW cells and BMDM exhibit differing expression levels of phagosomal proteins and phagocytic capacity (Guo et al., 2015), as well as differing transcriptional responses to LPS (Das et al., 2018) and murine novovirus (Levenson et al., 2018). We propose that thioglycolate-elicited peritoneal macrophages may better model the IGF-1 response of macrophages in injured muscle (compared to BMDMs) because both are recruited to the tissue via inflammation, a process that influences transcriptional responses to a wide range of stimuli (Netea, 2020). However, future work will need to confirm that ATRA-PLG induces IGF-1 in muscle, as well as the cellular sources of IGF-1.

We suspected the differing IGF-1 responses to ATRA among the macrophages studied might stem from differences in RAR protein levels. To our knowledge, the relative difference in RAR protein levels among these three commonly studied macrophages have not been directly compared. We found that RARβ was produced by all three cell types, with peritoneal macrophages having the highest level. In contrast, RARα and RARγ were detected only in RAW cells. Our data are consistent with the literature, which indicates that all three cell types express RARβ at either the protein or mRNA level (Ma et al., 2020; The Immunological Genome Consortium et al., 2012; Okabe and Medzhitov, 2014). Our data conflicts with one study that detected RARα protein in BMDMs from C57BL/6 mice (Garabuczi et al., 2023). This discrepancy might be due to our use of CD-1 mice; it is known BMDMs derived from different mouse strains exhibit differing gene expression profiles (Rabhi, 2013). Finally, we note that RARγ mRNA has been detected in peritoneal macrophages, but protein levels were not assessed in that study (The Immunological Genome Consortium et al., 2012). Taken together, our data support a model in which high levels of RARβ (peritoneal macrophages) or moderate levels of RARβ with RARα and/or RARγ (RAW macrophages) are needed for macrophages to produce IGF-1 in response to ATRA.

We developed a PLG microparticle system for extended release of ATRA (i.e., ATRA-PLG). The release profile was linear over the time period studied (days 1 to 14), which is consistent with previous studies that encapsulated ATRA into PLG microparticles (Giordano et al., 1993; O’Connor, 2019; Jeong, 2003). The extended release profile may be optimal for treating human muscle atrophy because recovery with exercise interventions takes at least 4 weeks (Elam, 2022) and reducing dosing frequency (which extended release affords) can increase patient compliance with a pharmacotherapy (Srivastava et al., 2013). Importantly, drug release profile can be modulated by changing the carrier properties such as polymer molecular weight (Isely et al., 2019; Spetz et al., 2021); thus, we predict the release profile can be tailored as needed for specific treatments in humans. Looking towards clinical translation, we envision ATRA-PLG could be delivered to debilitated muscle using ultrasound-guided delivery, which is already used in humans for localized musculoskeletal interventions around the hand, wrist, shoulder, and lower limbs (Dave et al., 2014; Orlandi, 2014; Orlandi, 2016; Messina, 2016; Tortora, 2021).

The drug loading for ATRA-PLG was 78 μg/mg, which is higher than previous formulations, which ranged from 20 to 50 μg/mg (Giordano et al., 1993; O’Connor, 2019; Jeong, 2003). Drug loading into PLG microparticles using single emulsions is dependent on several factors including PLG composition and molecular weight, weight ratio of ATRA and PLG in the oil phase, and the solvent used in the oil phase (Wischke and Schwendeman, 2008). This study and previous work (Giordano et al., 1993; O’Connor, 2019; Jeong, 2003) all used DCM and 50:50 PLG with similar molecular weights. We propose that the higher drug loading in this study is linked to an optimum weight ratio of ATRA and PLG (1:10) combined with a high concentration of ATRA in the oil phase (5 mg/mL). We selected these parameters empirically to yield 2–3 μm diameter particles with high loading of ATRA. Microparticles within this size range are readily internalized by macrophages (Champion et al., 2008) and may support extended drug release within macrophages for the modulation of intracellular receptors. We used the fluorescent molecule Coumarin-6 to visualize small molecule release from PLG particles internalized by BMDM. Coumarin-6 has LogP and molecular weight values that are similar to ATRA and may model its release from the particles (LogP_C6_ 4.9, LogP_ATRA_ 6.3; MW_C6_ 350, MW_ATRA_ 300). Particle treatment of BMDMs led to internalization and long-term retention of the particle without signs of toxicity. Taken together, these results suggest the particles developed herein may be used to achieve long-term drug delivery to macrophages in vivo. Also, we propose that lipophilic drugs, such as ATRA, may be able to exit the macrophage by crossing the cell membrane and act on adjacent cells such as muscle progenitor cells (in the case of ATRA-PLG delivery to injured muscle).

We used the C2C12 myotube formation assay to assess ATRA-PLG as a muscle pharmaceutic that might act indirectly on muscle growth through macrophages. We found that RAW conditioned media or ATRA (released from ATRA-PLG) augmented C2C12 myotube formation to the same level. Also, the effect of RAW conditioned media was enhanced when RAW cells were pre-treated with ATRA-PLG, and a neutralizing antibody indicated IGF-1 was a causative factor. Our data are consistent with the literature, which indicates that C2C12 myotube formation is augmented by either macrophage conditioned medium (Dumont and Frenette, 2013; Dumont and Frenette, 2010) or “free” ATRA (Zhu, 2009; Alric et al., 1998; Chen and Li, 2016; Li et al., 2020). The novelty of our study is the demonstration that pretreatment of macrophages with ATRA-PLG leads to a more potent “conditioned media” for C2C12 myotube formation, and IGF-1 content appears to be the cause. We have considered that ATRA-PLG may not increase macrophage IGF-1 in muscle after localized delivery (i.e., a BMDM-type response). However, the ability of ATRA (released from ATRA-PLG) to induce C2C12 myotube growth independent of macrophages still strongly supports future rodent testing of this promising pharmaceutic in the treatment of injured or atrophied muscle. Also, it will be important to confirm ATRA-PLG stability under physiological conditions over time frames that reflect the proposed treatment regimen.

## 5. Conclusion

All-trans retinoic acid (ATRA) induces insulin-like growth factor (IGF)-1 secretion by RAW 264.7 macrophages and thioglycolate-elicited peritoneal macrophages, but not bone marrow derived macrophages. Differential expression of retinoic acid receptor isoforms may account for these differences. Towards leveraging these findings for muscle therapy, the ATRA-PLG maintains ATRA bioactivity and enables its extended release over the time frame of weeks. These attributes may improve the effectiveness of a pharmacotherapy by increasing bioavailability of ATRA and patient compliance with the dosing schedule. ATRA-PLG particles are readily internalized by macrophages and do not induce toxicity over the course of weeks. Also, ATRA-PLG robustly enhances myotube formation through direct action of ATRA on myoblasts and indirectly through macrophage IGF-1. Given that both macrophages and myoblasts are present in injured muscle at high levels, the work strongly supports further testing of this promising pharmaceutic using rodent models of muscle regeneration and growth.

## Supplementary Material

MMC1

## Data Availability

Data will be made available on request.
