# Supplementary material for: A microparticle delivery system for extended release of all-trans retinoic acid and its impact on macrophage insulin-like growth factor 1 release and myotube formation
Source: Int J Pharm. Author manuscript; Available in PMC 2025 Jan 7. (PMC11706047; doi:10.1016/j.ijpharm.2024.124821)
Supplement: MMC1 [file NIHMS2030873-supplement-MMC1.docx]

**Supplementary Material Document**

A microparticle delivery system for extended release of all-trans retinoic acid and its impact on macrophage insulin-like growth factor 1 release and myotube formation

Authors and Affiliations

Candice V. Cheung^a^, Kidochukwu J. Atube^b^, Nicholas A. Colonna^a^, Griffin J. Carter^a^, Tristan Marchena^a^, Samantha McCarthy^a^, Kelsey E. Krusen^a^, Richard S. McCain^c^, Norma Frizzell^c^, R. Michael Gower^a,b,d*^

^a^Biomedical Engineering Program, University of South Carolina, Columbia, SC 29208, USA

^b^Department of Chemical Engineering, University of South Carolina, Columbia, SC 29208, USA

^c^Department of Pharmacology, Physiology & Neuroscience, School of Medicine, University of South Carolina, Columbia, SC, 29209, USA

^d^Veterans Affairs Medical Center, Columbia SC, 29209, USA

*Corresponding Author

Prof. R. Michael Gower

Department of Chemical Engineering

University of South Carolina

Swearingen Engineering Center Room 2C21

301 Main Street Columbia, SC 29208

Phone: 803-777-1541

Fax: 803-777-0973

Email: [gowerrm@mailbox.sc.edu](mailto:gowerrm@mailbox.sc.edu)

**
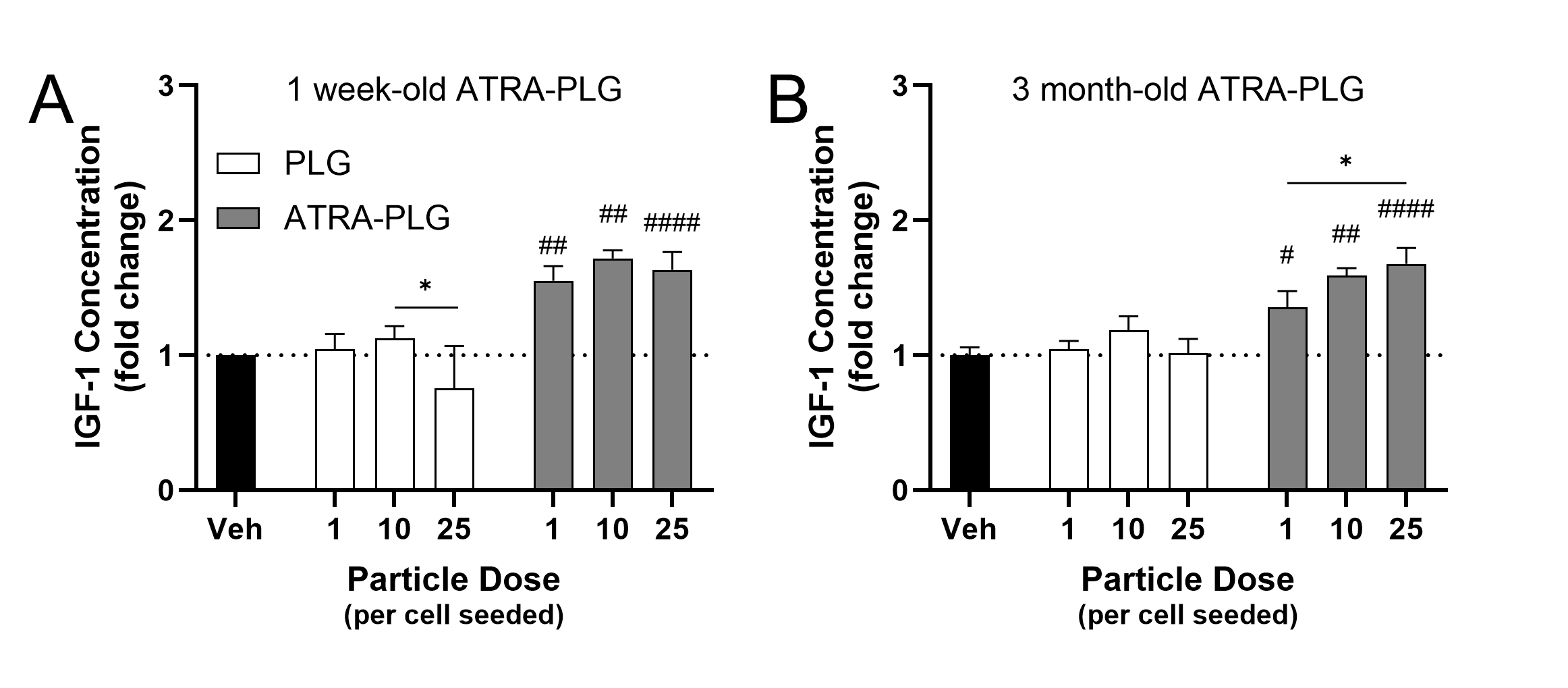
**

**Supplementary Figure 1: Stability of ATRA-PLG particles.** (A) RAW macrophages were treated with ATRA-PLG or PLG particles that were stored at room temperature under vacuum and in the dark for one week. (B) Three months later, particles from the same batch, under the same storage conditions, were assayed a second time**.** For both experiments, particle treatment was for 24 hours at doses of 1, 10, or 25 particles per cell seeded. IGF-1 concentration in cell media was measured by ELISA. Two-way ANOVA with Tukey’s multiple comparison was conducted between PLG and ATRA-PLG with particle type and dose as sources of variation. #, ##, and #### indicate p < 0.05, 0.01, and 0.0001 versus PLG particles at the equivalent dose. * indicates p < 0.05 as indicated. Data is from one experiment with 3 technical replicates per condition. Data are mean ± SD.


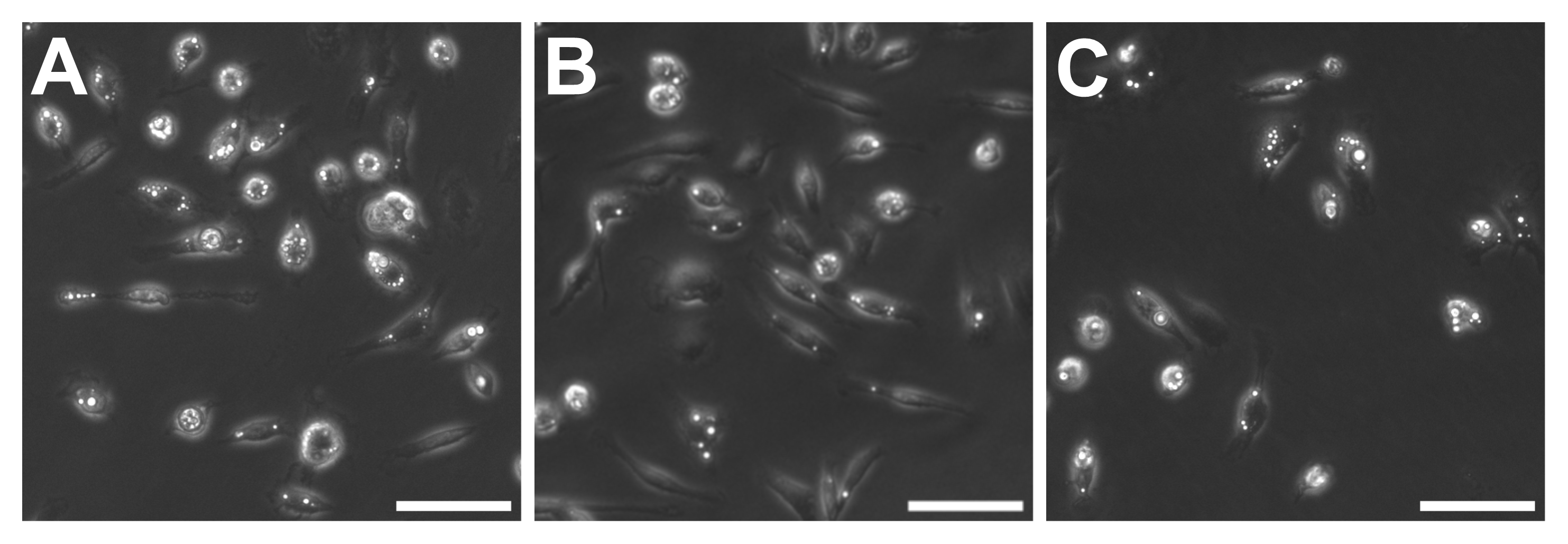
**Supplementary Figure 2:** Three representative images (A, B, C) of ATRA-PLG particles in BMDMs after 12 days of culture. Scale bar = 50 µm.

| 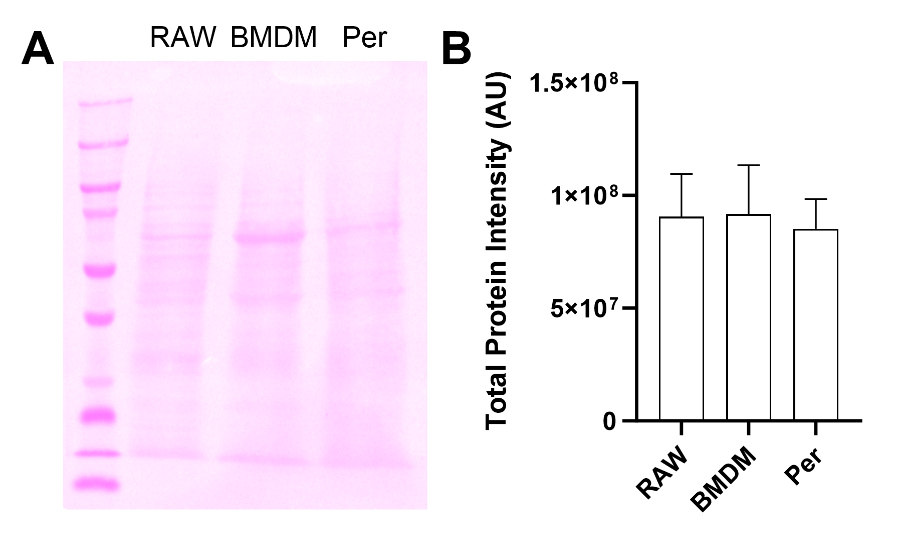 | **Supplementary Figure 3: Total protein normalization of macrophages.** (A) Representative Ponceau-S stain of membrane after western blotting. (B) Relative intensity of ponceau stain. One-way ANOVA was conducted (no significance found). n = 4 independent experiments. Data are expressed as means ± SD. |
| --- | --- |

| 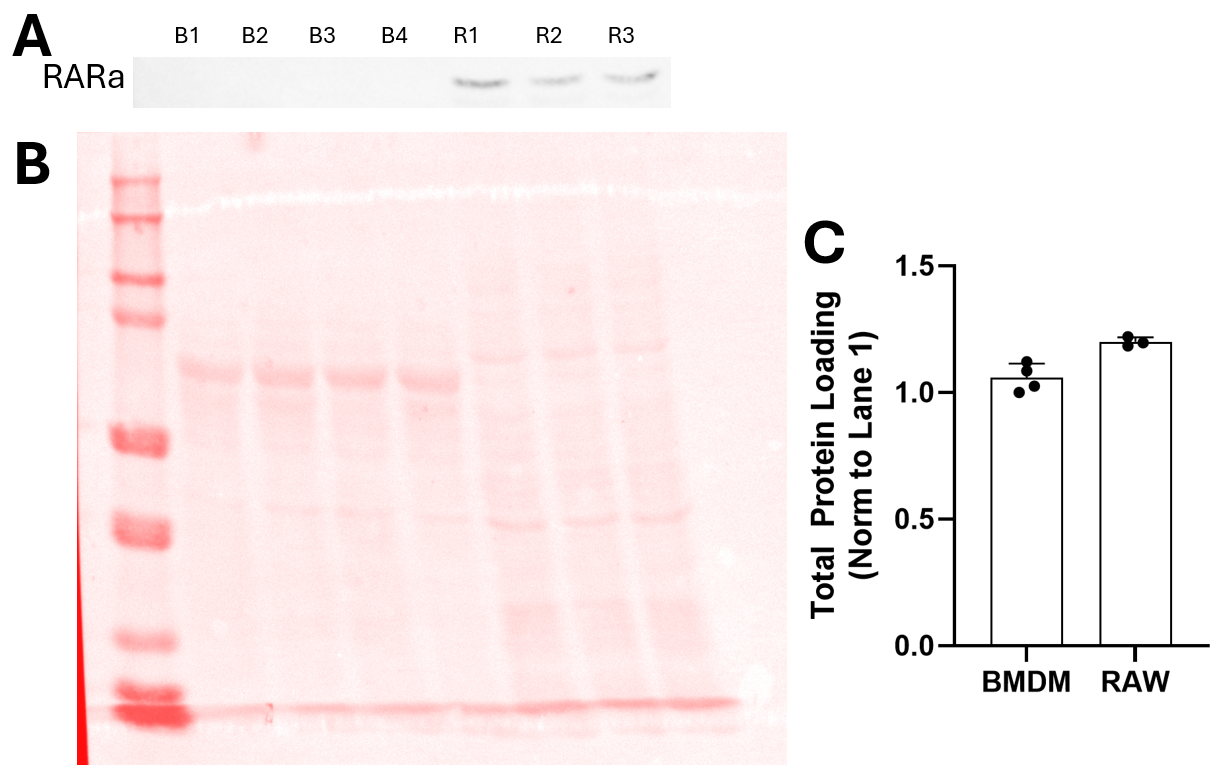 | **Supplementary Figure 4: RARα in RAW macrophages and BMDM probed with a second antibody clone.** Representative western blot (A) and Ponceau stain (B) of BMDMs (B1-4) and RAW macrophages (R1-3) using a Santa Cruz RARα antibody sc-515796. (C) Relative intensity of ponceau stain. Student’s t-test was conducted (no significance found). n = 2 independent experiments. Data are expressed as means ± SD. |
| --- | --- |
